# Research progress and clinical application of biomarkers in neuropathic pain

**DOI:** 10.1186/s10194-026-02489-7

**Published:** 2026-08-05

**Authors:** Leibing Zhang, Haocheng Zhuang, Mingkun Ma, Shuyuan Xian, Wangzheqi Zhang, Hongda Bi, Shizhao Ji, Runzhi Huang

**Affiliations:** 1 Department of Plastic Surgery, Changhai Hospital, No. 168 Changhai Road, Yangpu District, Shanghai, 200433 China; 2 Department of Burn Surgery, Changhai Hospital, No. 168 Changhai Road, Yangpu District, Shanghai, 200433 China; 3 Department of Anesthesiology, Changhai Hospital, No. 168 Changhai Road, Yangpu District, Shanghai, 200433 China

**Keywords:** Neuropathic pain, Biomarkers, Multi-omics technology, Machine learning, Precision diagnosis and treatment

## Abstract

Neuropathic pain (NP) is a subtype of refractory chronic pain that currently presents three major difficulties in diagnosis and therapy in that clinicians must depend on patient complaints for the diagnosis, physicians find it difficult to recognize the disease early, and the evaluation of efficacy is slow. This review surveys the research on NP biomarkers conducted to date and aims to provide a scientific basis and reference for the exact diagnosis and treatment of NP. Studies indicate that the core pathological mechanism producing NP and its changes is the pathological plastic changes which have occurred in the sensory nervous system and that distinct etiologies of NP are associated with unique pathological mechanisms. NP biomarkers exist in such facets of investigation as biochemical, electrophysiological, imaging, genetic and epigenetic studies. Each category of biomarkers can provide specific utility for the diagnosis and prognostic assessment of NP. However, the high heterogeneity of NP limits the clinical translatability of individual biomarkers. Therefore, a multimodal integration of biomarkers is required for NP diagnosis and treatment, utilizing strategies including multicenter clinical research and therapeutic interventions, integrated multi-omics data analysis, to progressively develop an increasingly precise diagnostic and therapeutic system. These efforts will progressively facilitate the transition of the NP diagnostic and therapeutic model from traditional empirical medicine to a data-driven paradigm.

## Clinical trial registration number

Not applicable.

## Introduction

NP represents an important category of clinically resistant pain produced directly by injury to, or pathological change in, the somatosensory nervous system, characterized principally by spontaneous pain, hyperalgesia, allodynia and other accompanying phenomena. This condition seriously disturbs the quality of life of the patients who suffer from it and creates a serious tax on the welfare of the community [1]. The epidemiologic data available show that the prevalence of NP is approximately 7%-10% in the general population worldwide [2]. But the currently available diagnostic and therapeutic means for NP have significant drawbacks. They are largely based on patients’ subjective complaints or neurological physical examinations that rely heavily on these complaints. There is no support from objective indicators for these means. They may also be influenced by psychological factors. The early pathological changes that characterize these conditions usually escape detection due to their insidious nature. This prevents any chance of implementing appropriate interventions. In addition, the efficacy assessment of the obtained results depends on follow-up. It cannot indicate the real quantitative condition experienced by patients in the interim [3].

Biomarkers, as objectively detectable indicators associated with pathological processes, are the main weapons for solving the above clinical problems. They can not only clarify the pathological mechanism of NP, but also can be used for screening high-risk groups of patients with NP, early diagnosis of the disease, and prediction of treatment results [4]. In recent years, with the rapid development of molecular biology, electrophysiology, imaging and multi-omics technology, significant progress has been made in the research of NP related biomarkers. Studies have confirmed that neuroinflammation, oxidative stress, dysregulation of ion channels and epigenetic modification are the main pathways of NP occurrence and development [5–9]. Thanks to the technical breakthroughs, the application of high-throughput sequencing technology, functional magnetic resonance imaging (fMRI) and machine learning algorithms, the analytical way of NP multidimensional integration has been facilitated [10–12].

Nonetheless, numerous obstacles remain in research on NP biomarkers. First, the diagnostic usefulness of a single biomarker is very limited, and their specificity and sensitivity must be improved [13–15], and thus the necessity to develop a composite biomarker system is urgent. Second, NP exhibits extreme disease heterogeneity, as the pathogenesis varies across different etiologies and distinct biomarkers are therefore required for different etiological subtypes [16]. Finally, the clinical translation cycle is fairly protracted, since the verification process from the discovery of the biomarker in basic research to clinical use is exceedingly time-consuming [17].

Hence, this review systematically sums up the current research status of NP biomarkers, explores the associations between different kinds of biomarkers and the pathological mechanisms of NP, critically discusses the advantages and disadvantages of existing researches, explores the prospect of the application of multi-omics integration technology and machine learning algorithm in NP biomarkers. This review aims to provide systematic references for the basic research and clinical translation of NP biomarkers.

## Pathological mechanisms of neuropathic pain

### Common mechanisms

**Fig. 1:**
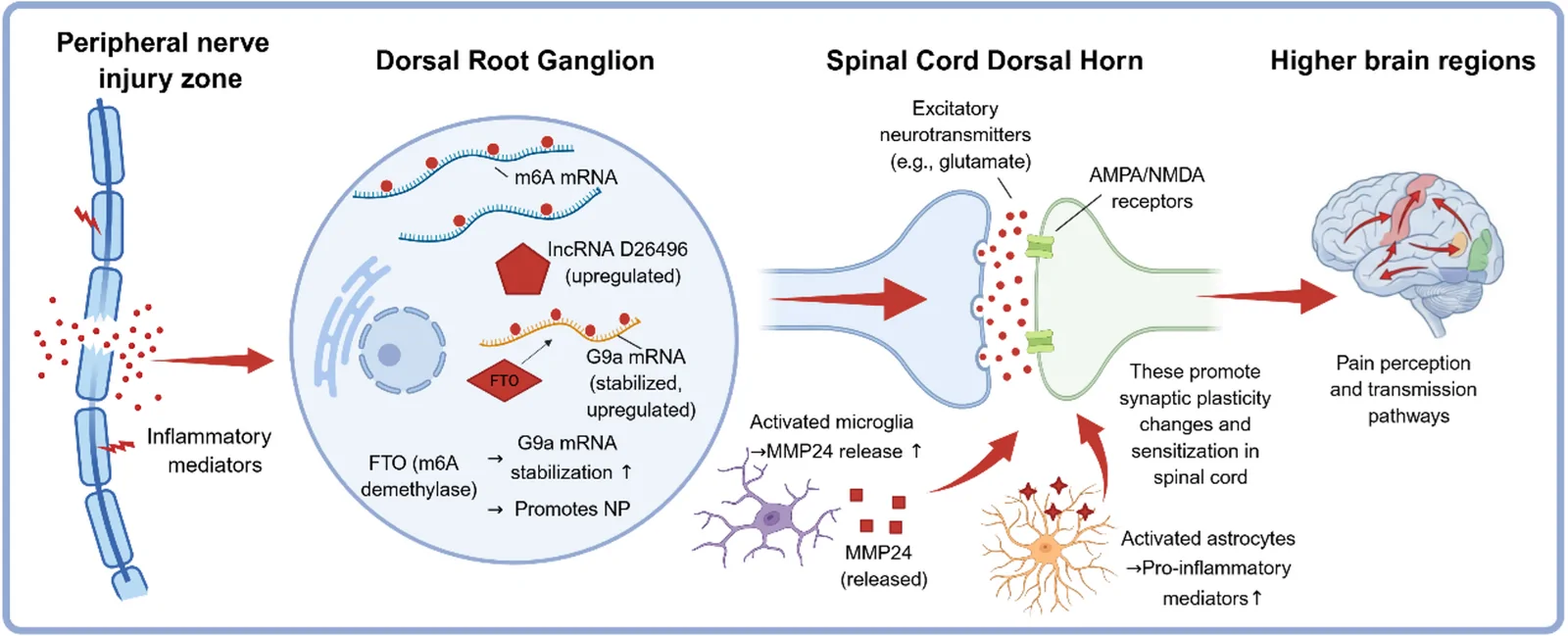
Pathological mechanisms and biomarker localization in the pathogenesis of neuropathic pain. This figure depicts the spatial and molecular progression of neuropathic pain signaling, encompassing the continuum from peripheral nerve injury to central sensitization and cortical pain perception, as well as the localization of key pro-nociceptive biomarkers

The pathological mechanisms of NP are complex and diverse, and have not been fully elucidated. Studies have confirmed that its core mechanism is closely linked to pathological plastic changes in the sensory nervous system that involves bidirectional interactions between the peripheral nervous system (PNS) and the central nervous system (CNS) [18]. At the ion channel level, peripheral sensory neurons exhibit abnormal upregulation of the expression of sodium and calcium channels and downregulation of the expression of potassium channels. This leads to increased neuronal excitability and ectopic discharge, thereby inducing hyperpathia, hyperalgesia, and spontaneous pain [19]. Within the CNS, plastic alterations occur at all levels of pain transmission (from the dorsal horn of the spinal cord to the primary sensory cortex) that initial changes are functional (e.g., enhanced excitatory synaptic transmission), whereas later changes are morphological (e.g., increased number of excitatory synapses and decreased number of inhibitory synapses), ultimately resulting in the remodeling of pain signal processing patterns [20]. During neuroinflammation and immune activation, activated neuroglial cells (e.g., microglia and astrocytes) release proinflammatory cytokines such as TNF-α and IL-1β, which contribute to the development of chronic pain by regulating ion channel expression and synaptic plasticity. Moreover, peripheral and central nerve injuries impair the blood-nerve barrier and blood-brain barrier, leading to immune cell infiltration and further exacerbation of the inflammatory response [21]. Plastic alterations in the pain modulation system are characterized primarily by weakened function of the descending inhibitory system and enhanced activity of the descending facilitatory system, ultimately resulting in the amplification of pain signals [22]. In addition, structural and functional alterations in brain regions such as the hippocampus and medial prefrontal cortex induce cognitive impairment and emotional abnormalities, and in turn cognitive dysfunction further enhances pain perception by interfering with the pain modulation system [23] (Fig. 1).

### Etiology-specific mechanisms

The specific mechanism of peripheral diabetic neuropathy (PDN) encompasses multiple interacting pathological processes that hyperglycemia activates the polyol pathway and protein kinase C (PKC) signaling pathway and drives the accumulation of advanced glycation end products (AGEs), thereby causing ischemia-hypoxia and oxidative damage to nerve tissue, among which small-diameter nerve fibers are particularly vulnerable. Decreased levels of neurotrophic factors induce neuronal apoptosis and initiate axonal transport disorders and diabetic microangiopathy diminishes intraneural blood flow, further exacerbating ischemic damage.

The pathogenesis of postherpetic neuralgia (PHN) can be summarized as the varicella-zoster virus (VZV) remaining latent in the sensory ganglia, and upon reactivation, it directly causes neuronal degeneration, demyelination, and axonal fragmentation, ultimately forming neuroma-like structures. Viral infection triggers neuroinflammation, thereby activating glial cells to release proinflammatory factors, which in turn recruit immune cells and induce chronic ganglionic inflammation and sensitization of pain conduction pathways. Inflammation also activates the sympathetic nervous system and the norepinephrine released by this system further amplifies the transmission of pain signals through sympathetic-sensory nerve crosstalk [24–26].

Central neuropathic pain (CNP) frequently develops following spinal cord or traumatic brain injury, and its pathological processes have several key characteristics, including enhanced neuronal reactivity in the spinal dorsal horn (i.e., central sensitization), a reduction in the number of inhibitory interneurons and excessive proliferation of excitatory synapses, and all these features contribute to the remodeling of pain signal processing patterns. Glial scarring at the injury site impairs nerve regeneration and induces demyelinating lesions, ultimately leading to abnormal electrical signal conduction in nerve fibers. Furthermore, spinal cord injury can damage the brainstem-spinal cord descending inhibitory pathways, compromising the capacity of the CNS to modulate pain [18, 22, 27, 28].

### Sensory phenotypic heterogeneity and underlying mechanisms

As proposed by Baron and colleagues, neuropathic pain can be classified into distinct sensory phenotypes based on quantitative sensory testing profiles, which reflect different pathophysiological mechanisms rather than simply different etiologies [16]. Three major phenotypes have been identified, including the irritable nociceptor phenotype characterized by preserved small-fiber function and pronounced hyperalgesia/allodynia, the non-irritable nociceptor phenotype marked by impaired nociceptor function and reduced sensory detection, and the deafferentation phenotype with severe loss of afferent input and central disinhibition.

Accumulating evidence indicates that differential biomarker signatures underlie these sensory phenotypes. In the irritable nociceptor phenotype, electrophysiological biomarkers show preserved or enhanced nociceptive evoked potential amplitudes with shortened latencies, and biochemical biomarkers reveal elevated proinflammatory cytokines (TNF-α, IL-1β) and increased sodium channel function, reflecting peripheral sensitization. In contrast, the non-irritable and deafferentation phenotypes present with reduced or absent laser-evoked potentials, decreased intraepidermal nerve fiber density, and imaging evidence of spinal or thalamic deafferentation remodeling. Phenotype-specific biomarker patterns may explain why patients with the same etiology but different sensory profiles exhibit distinct treatment responses, supporting the value of biomarker-guided phenotypic stratification for personalized therapy.

From a clinical symptom perspective, neuropathic pain consists of three distinct components, namely continuous spontaneous pain (persistent burning, aching or tingling sensations), paroxysmal spontaneous pain (sudden shooting, electric shock-like or stabbing attacks), and evoked pain (pain triggered by normally non-noxious or mildly noxious stimuli, i.e., allodynia and hyperalgesia). Each component is associated with partially distinct pathophysiological mechanisms and corresponding biomarker signatures. Continuous spontaneous pain is primarily associated with ongoing ectopic discharge of damaged primary afferents and central disinhibition. Electrophysiologically, it correlates with abnormal spontaneous firing of DRG neurons and altered resting-state EEG oscillatory activity in the θ-low α frequency band. Biochemically, sustained elevation of proinflammatory cytokines (TNF-α, IL-1β) and tonic activation of sodium channels (Nav1.7, Nav1.8) contribute to the maintenance of continuous pain. Paroxysmal spontaneous pain is linked to high-frequency burst firing of hyperexcitable nociceptors and ephaptic transmission between damaged nerve fibers. It is more closely associated with ion channel dysfunction (e.g., gain-of-function Nav1.7 mutations) and sympathetic-sensory coupling, particularly in conditions such as trigeminal neuralgia and postherpetic neuralgia. Evoked pain (allodynia and hyperalgesia) reflects central sensitization of spinal dorsal horn neurons and enhanced synaptic transmission. It correlates with biomarkers of spinal glial activation (TSPO upregulation), increased excitatory neurotransmission, and wide dynamic range (WDR) neuron hyperexcitability. Evoked pain is also the component most commonly measured in animal models, which explains why biomarkers related to central sensitization are most frequently identified in preclinical studies. The Neuropathic Pain Symptom Inventory (NPSI) is a validated patient-reported outcome instrument that quantifies the intensity of these distinct pain dimensions, enabling standardized phenotypic stratification of NP patients.

## Classification and application of biomarkers for neuropathic pain

NP biomarkers can be classified into four types, each corresponding to a specific link in the pathological mechanisms of NP, on the basis of different detection methodologies. Given that a single biomarker fails to comprehensively capture the pathological features of the NP, the combined application of multiple biomarkers is necessary to enhance diagnostic efficacy.

From a clinical translation perspective, NP biomarkers differ substantially in detection accessibility and invasiveness. Non-invasive biomarkers, including serum/plasma biochemical molecules, surface electrophysiological recordings, and extracranial imaging, are most suitable for large-scale screening and longitudinal follow-up. In contrast, biomarkers requiring cerebrospinal fluid sampling, nerve biopsy, or invasive electrophysiological monitoring exhibit higher diagnostic precision but limited clinical accessibility, and are typically reserved for differential diagnosis of complex cases.

### Biochemical biomarkers

Biochemical biomarkers directly reflect the pathological progression of NP by detecting molecular alterations in bodily fluids and are among the biomarkers that currently hold the highest potential for clinical translation. These biomarkers fall mainly into three categories including neuroinflammation-related biomarkers, metabolism-related and oxidative stress-related biomarkers, and noncoding RNA biomarkers.

#### Neuroinflammation and immunity-related biomarkers

Neuroinflammation is a core pathological component of the NP but differences in inflammation-initiating mechanisms across distinct etiologies lead to the specific distribution of biomarkers. In PDNs, hyperglycemia, oxidative stress, and AGEs induce the activation of signaling pathways such as the TLR and NF-κB pathways, thereby driving the release of cytokines, including TNF-α, IL-1β, and IL-6. This triggers immune cell infiltration, nerve fiber damage, and hyperalgesia, thereby forming a vicious “inflammatory-destructive” cycle [29]. TLR4 is a vital regulatory molecule in PDN-related inflammation. Its expression levels in monocytes from PDN patients are 5-fold higher than those in diabetic patients without neuropathy and its expression is positively correlated with the levels of TNF-α and IL-6. Inhibiting TLR4 activity not only alleviates NP-related symptoms but also effectively mitigates the inflammatory response [30, 31]. As a core proinflammatory cytokine, TNF-α is positively correlated with serum TNF-α levels and pain intensity, the degree of reduced nerve conduction velocity, and the extent of nerve demyelination in PDN patients, and inhibiting TNF-α function can significantly improve nerve function [32–35]. Moreover, TNF-α can act synergistically with IL-1β to activate the NF-κB pathway, further promoting vascular endothelial dysfunction and immune cell infiltration. The mRNA and protein levels of IL-1β are significantly elevated in both the serum and nerve tissues of PDN patients and are closely associated with neuronal dysfunction and chronic pain [35, 36]. In contrast to the aforementioned proinflammatory cytokines, IL-6 levels are negatively correlated with insulin resistance, vascular complications, and nerve fiber damage in PDN patients, and high-dose IL-6 can improve nerve blood flow [37–39]. Additionally, MCP-1 can be induced by TNF-α and IL-1β, which further exacerbates the chronic inflammatory response in PDNs by recruiting monocytes to infiltrate nerve tissues [40].

The immune system plays dual roles in pain regulation. Immune cells can induce NP by releasing proinflammatory cytokines to activate nociceptors and can also alleviate pain by releasing anti-inflammatory cytokines and endogenous opioids or by mediating nerve regeneration [41]. Among these, IL-10 exerts an analgesic effect by acting directly on nociceptors and inhibiting the synthesis and release of proinflammatory cytokines [42, 43] and IL-4 promotes macrophages to synthesize endogenous opioids and polarize toward a pro-repair phenotype, thereby indirectly relieving pain [44, 45].

In PHN, VZV-specific cellular immunity mediates viral clearance through the activation of specific T cells. If the body undergoes aging or immune function is suppressed, this viral clearance capacity decreases, thereby promoting VZV reactivation [46]. VZV-specific T cells selectively infiltrate and then persist in rash-affected regions. If the function of these cells is compromised, VZV DNA can be continuously detected in the patient’s saliva and the risk of developing PHN is significantly increased [47, 48]. IFNs act as antiviral defenses and immune regulatory factors. Inherited defects in their downstream signaling pathways increase the body’s susceptibility to acute VZV infection. IFN can inhibit VZV replication in neurons. Local administration of IFN-α2b into the DRG can reduce pain and decrease the incidence of PHN. Furthermore, the expression levels of IFN-A1/13 and IFN-A2 in PHN patients are greater than those in healthy controls and those of IFN-4 and IFN-10 are greater than those in patients with herpes zoster, suggesting that these IFN subtypes may serve as potential diagnostic biomarkers for PHN [49–52].

It should be emphasized that neuroinflammation in neuropathic pain differs conceptually from systemic acute inflammatory pain. The core pathological process in NP is localized glial activation, immune cell infiltration within damaged nerve tissue, and inflammatory mediator release in the peripheral and central nervous system, which drives neuronal sensitization rather than serving as a non-specific systemic inflammatory response. The profile of inflammatory biomarkers varies across NP etiologies. Inflammatory signatures are most prominent in metabolic and infectious neuropathies (e.g., PDN, PHN), while in traumatic or central neuropathic pain, inflammation is more restricted to the local neural tissue and may not be readily detectable in peripheral blood. Most neuroinflammation and immunity-related biomarkers can be detected via peripheral venous blood sampling, which is minimally invasive and widely applicable in routine clinical practice.

#### Metabolism and oxidative stress biomarkers

Oxidative stress is a core pathological feature of PDN, and related biomarkers exhibit clear etiology-specific targeting properties. Under hyperglycemic conditions, glucose undergoes nonenzymatic reactions with amino acids to form AGEs, which in turn induce glycotoxic effects. Specific receptors for AGEs are present on the surfaces of neurons, Schwann cells, and vascular endothelial cells, and when AGEs bind to these receptors, they activate NADPH oxidase thereby promoting the generation of reactive oxygen species (ROS). ROS regulate gene expression through NF-κB-mediated signaling pathways which not only induce inflammatory responses but also disrupt the balance between cell survival and apoptosis and this toxic effect also manifests itself in the nervous system [53–56]. Additionally, AGE receptor signaling can further enhance TRPV1-mediated calcium signaling, inducing painful neuropathy [57]. MG, an intermediate product of nonenzymatic glycosylation, plays a vital role in diabetic complications. Under diabetic conditions, MG accumulates in vivo and reacts with proteins to form AGEs. However, mice overexpressing glyoxalase 1 do not develop degenerative diabetic neuropathy [58]. MGs can bind Nav1.8 in sensory neurons, enhancing neuronal excitability, and induce pain by activating TRPA1 channels and triggering an integrated stress response [59, 60], moreover, significantly elevated plasma MG levels are a risk factor for neuropathy in patients with type 2 diabetes mellitus [61].

Studies investigating mitochondrial respiratory dysfunction have yielded key findings across both inflammatory pain and neuropathic pain models. In the carrageenan-induced transient inflammatory pain model, the oxygen consumption rate (OCR) of lumbar DRG neurons is significantly reduced during the peak pain period, and returns to normal following pain relief. In a complete Freund’s adjuvant (CFA)-induced persistent inflammatory pain model, the expression levels of multiple proteins in the mitochondrial electron transport chain (mETC) in DRG neurons are reduced, indicating inhibited mitochondrial respiratory function. Notably, consistent mitochondrial respiratory dysfunction has also been verified in classic neuropathic pain models, with comparably reduced oxidative phosphorylation levels detected in the spinal cord tissues of CFA-induced inflammatory pain rats and chronic constriction injury (CCI)-induced neuropathic pain mice. This finding suggests that mitochondrial respiratory impairment is a shared mechanism underlying both inflammatory pain and neuropathic pain, albeit triggered by different upstream pathological stimuli [62–64].

Approximately 0.2%–2.0% of the oxygen consumed by mitochondria is reduced to superoxide anions (O₂⁻), which can be further converted into ROS such as hydrogen peroxide (H₂O₂) and hydroxyl radicals (OH⁻) [65]. When ROS production is excessive or the antioxidant defense system is compromised, the balance between mtROS production and clearance is disrupted, thereby leading to oxidative stress [66]. Among the contributing factors, mETC dysfunction is the main driver of mtROS production and additionally, reduced mitochondrial membrane potential and compromised calcium buffering capacity may also increase mtROS production and disrupt redox balance [66–68]. Owing to their high energy metabolism requirements and high content of oxidizable molecules, neurons are more sensitive to mtROS-induced oxidative stress [66, 69, 70]. For example, antimycin A can activate TRPA1 receptors by inducing mtROS production thereby increasing the excitability of sensory neurons. Following treatment with ROS donors, the excitability of spinal neurons is significantly enhanced, and the intrathecal injection of ROS donors can induce mechanical hyperalgesia in rats. Furthermore, elevated ROS production in the spinal cord can promote pain sensitization by inhibiting the firing of inhibitory neurons [71–74].

The high energy demand of neurons depends on mitochondrial ATP production with approximately 90% of ATP generated through oxidative phosphorylation and impairment of this process leads to reduced dendritic synaptic plasticity which in turn causes neuronal damage or even death [75, 76]. Disruption of neuronal mitochondrial respiratory function affects sensory signal processing and induces pain, and for example, intrathecal injection of mETC complex Ⅰ or Ⅲ inhibitors into normal mice can induce mechanical allodynia, and human mETC complex Ⅰ deficiency is associated with muscle pain [77, 78]. Additionally, mitochondrial respiratory dysfunction can indirectly promote pain development. For example, disruption of genes related to mETC complex Ⅳ activity in mouse DRG neurons leads to reduced ATP production and an elevated ADP/ATP ratio, and ADP then induces mechanical and thermal hyperalgesia by activating the purinergic receptor P2Y1 [79]. Pyruvate enters mitochondria to participate in the tricarboxylic acid (TCA) cycle under normoxic conditions, while it is converted to lactic acid under hypoxic conditions. As a signaling molecule, lactic acid is involved in various physiological processes and it exerts anti-inflammatory effects under certain conditions. For example, lactic acid accumulation in dendritic cells can promote the transition of inflammatory phenotypes by regulating IL-10 secretion [80]. However, under other conditions, lactic acid can inhibit the migratory capacity and cytotoxicity of CD8^+^ T cells, promote the proliferation of regulatory T cells [81], enhance NF-κB pathway-mediated immune responses and inflammatory cascades in Th17 cells [82], stimulate the NF-κB/IL-8 pathway in endothelial cells, induce ROS production and disrupt the blood-brain barrier [83]. From the perspective of pain regulation, the inflammatory effects of lactic acid and the induction of oxidative stress in peripheral tissues promote pain sensitization and, in the brain, and spinal cord, lactic acid that accumulates during chronic nerve injury can enter neurons and microglia and contribute to ATP and ROS production as a TCA cycle substrate. The immunomodulatory factors and ROS produced by microglia promote apoptosis and autophagy through cascade reactions, alter the stability and permeability of ion channels, and promote pain sensitization through synaptic membrane depolarization and synaptic remodeling [84, 85]. Metabolic and oxidative stress biomarkers such as AGEs, methylglyoxal and lactic acid can be quantified in serum or plasma through routine biochemical testing, representing highly accessible non-invasive indicators.

#### Regulation of inflammatory/metabolic pathways by noncoding RNAs

In addition to small-molecule metabolites and inflammatory factors, noncoding RNAs are capable of regulating the secretion of inflammatory factors and the expression of oxidative stress-related genes via posttranscriptional regulatory mechanisms. miRNAs regulate the expression of multiple genes by interacting with the 3’ untranslated regions (3’ UTRs) of mRNAs, thereby participating in immune and inflammatory reactions. miRNAs may be vital molecules that mediate changes in the expression profiles of receptors and mediators in the NP and have potential value in the screening of diagnostic biomarkers and the development of therapeutic targets [86–88]. In the study of gene regulatory systems evaluating miRNA expression profiles is highly important particularly when these profiles are combined with mRNA expression analysis and other genome-wide data and this integration further enhances their overall research value [89]. Currently, the identified miRNAs and their potential target molecules encompass multiple signaling pathways related to pain perception, and a variety of miRNA-based therapies have advanced to stages spanning from preclinical research to phase II clinical trials [90]. Studies have demonstrated that miRNAs are closely associated with the onset and progression of NP, thereby providing a novel perspective for identifying vital molecular pathways and biological processes involved in the onset and maintenance of chronic pain [91, 92]. For example, in patients with lingual nerve neuroma, the expression levels of miR-29a and miR-500 are significantly positively correlated with pain visual analogue scale (VAS) scores after lingual nerve injury [93], and stimulation by inflammatory mediators can induce changes in the miRNA expression profiles of DRG neurons, which in turn regulate the expression of pain-related genes, increase the excitability of DRG neurons, and induce hyperalgesia [94]. However, there is currently no consensus on miRNA-based characteristic molecular biomarkers for the prognostic assessment of NP and prediction of treatment response.

In the human genome, less than 2% of genomic sequences encode proteins, whereas approximately 76% of genomic regions are transcribed into RNA. Among these transcripts, lncRNAs are a class of nonprotein-coding transcripts longer than 200 nucleotides. Their transcription is mediated by RNA polymerase II and they can undergo posttranscriptional modifications such as splicing [95–97]. Bioinformatics analyses have demonstrated that lncRNA molecules harbor functionally active structural modules and that RNA editing and chemical modifications can significantly alter their structure and function [98–100]. On the basis of single-cell sequencing studies of mouse and human DRG neurons, functional classification of somatosensory neurons can be accomplished on the basis of gene expression profile characteristics [101, 102]. At the posttranscriptional level, lncRNA LINC01119 expression is significantly upregulated in the spinal cord tissues of rat NP models induced by selective injury to sciatic nerve branches (SNI). Intrathecal injection of short hairpin RNA (shRNA) targeting LINC01119 effectively silenced LINC01119 expression and significantly alleviated pain symptoms in model animals. Clinical studies have demonstrated that serum LINC01119 levels are significantly elevated in patients with PHN, suggesting that it may serve as a potential biomarker for NP [103]. Importantly, when noncoding RNAs are used as biochemical biomarkers the core detection index is alterations in their expression levels while RNA modifications in epigenetics focus on the chemical modification status of RNA molecules. Circulating noncoding RNAs (miRNAs and lncRNAs) in peripheral blood are stable and detectable via quantitative PCR, offering a non-invasive and cost-effective approach for clinical screening.

### Electrophysiological and imaging biomarkers

Electrophysiological biomarkers enable the quantification of nerve function abnormalities, whereas imaging biomarkers facilitate the visualization of neural structural and functional remodeling. Combining these two types of biomarkers facilitates the “functional-structural” dual assessment of the NP, making it particularly suitable for evaluating small-fiber injuries and central lesions. Electrophysiological and most imaging biomarkers are entirely non-invasive. Standard nerve conduction studies, evoked potentials and EEG can be performed at bedside or in outpatient electrophysiology rooms, while ultrasound and conventional MRI are widely available in secondary and tertiary hospitals.

#### Electrophysiological detection techniques

Electrophysiological detection techniques directly reflect the function of ion channels and conduction pathways by recording neural electrical activity, and serve as a core tool for the objective diagnosis of the NP. However, appropriate detection techniques must be selected on the basis of the location, nature, and type of lesion. Currently, standard sensory electrophysiological tests have been widely applied in clinical practice and among these tests NCS and SEPs which utilize non-noxious electrical stimulation primarily evaluate non-nociceptive pathways namely peripheral large myelinated Aβ fibers and their central dorsal column-medial lemniscal pathway. In addition, specific evaluation of the thermos-nociceptive system (peripheral Aδ fibers, C fibers, and their central spinothalamic tract) is also clinically crucial for NP diagnosis [104–106]. When interpreting electrophysiological results for NP diagnosis, clinicians must integrate subjective pain reports, pain thresholds, response times, and sympathetic skin responses (SSRs). The stimulation site should be the painful region, with comparisons made to contralateral or adjacent unaffected anatomical areas [104]. Nevertheless, tests such as NCS or SEPs often yield normal results in patients with lesions involving only or primarily small fibers or the spinothalamic system, whereas these patients are precisely the high-risk population for developing NP, including those with small-fiber neuropathy and lesions in the spinal cord, brainstem, and other CNS regions [106–108]. Therefore, employing appropriate stimulation approaches to evaluate the thermos-nociceptive pathway is critical for diagnosing this patient population. Among the available techniques, laser-EPs and CHEPs are the most clinically valuable for identifying small-fiber lesions or spinothalamic tract lesions and significant abnormalities in these two techniques when painful regions are stimulated can serve as electrophysiological markers of the NP [109–111]. Electrophysiological studies also provide a foundation for the prognostic assessment of NP risk. For example, during surgical procedures, including thoracotomy, rhizotomy, and spinal tumor resection, intraoperative neurophysiological monitoring of sensory and motor potentials can effectively detect potential nerve damage that could result in postoperative NP, thereby offering critical guidance for clinical intervention [112–114]. A study of 42 consecutive patients with thalamic stroke demonstrated that combined assessment that utilizes laser-EPs and MRI to evaluate afferent fiber involvement in the thalamic spinothalamic tract achieved a classification sensitivity of 93% and a positive predictive value of 87% for patients with central thalamic pain [115]. Additionally, abnormal cortical functional alterations can be detected in patients with subacute spinal cord injury prior to NP onset, offering a potential basis for screening patients at high risk of developing NP [116, 117].

WDR neurons in the deep spinal dorsal horn receive projections from afferent fibers activated by both noxious and non-noxious mechanical stimuli and include both interneurons and projection neurons [118, 119]. In addition to peripheral synaptic input these neurons also receive intraspinal and supraspinal signals so their activity is dually regulated by local excitatory and inhibitory inputs and the brain’s descending pain regulatory system. Under pathological pain conditions, the excitability of peripheral or central nociceptive neurons is significantly enhanced, and WDR neurons exhibit characteristic changes, such as elevated spontaneous firing frequency and expanded receptive fields [120–122]. Among these changes elevated spontaneous firing of WDR neurons may be an important cause of spontaneous pain and ectopic discharge of damaged primary sensory afferent fibers can further increase the excitability and overall activity of WDR neurons and the elevated firing frequency of WDR neurons in pathological pain may be associated with increased activity of multiple nociceptive afferent fibers for example in rat nerve injury models the firing frequency of WDR neurons only returns to baseline when the activity of all nociceptive afferent fibers is completely blocked [123–125]. Notably, alterations in spontaneous firing of WDR neurons may be independent of peripheral input signals, but the regulatory mechanisms by which different types of afferent signals modulate the spontaneous firing of WDR neurons in pathological pain require further investigation [126, 127].

In clinical practice, pain diagnosis and management rely primarily on patients’ subjective reports, with supplementation from techniques such as sensory threshold measurement, electrophysiological potential recordings, and neuroimaging assessment. However, these techniques require the application of painful stimuli, resulting in their correlation with evoked pain mechanisms being significantly stronger than that with persistent spontaneous pain. For the assessment of spontaneous pain, resting-state recording methods (e.g., EEG) are more clinically appropriate. Effective pain biomarkers should be founded on the identification of pain-related brain activity patterns, which will help enhance diagnostic objectivity and prognostic accuracy [128]. Studies have demonstrated that the intensity of persistent NP is positively correlated with EEG activity in the θ-low α frequency band (4–10 Hz) and possibly with EEG activity in the high β frequency band across numerous brain regions but negatively correlated with EEG activity in the high α-low β frequency band in these regions. NP patients’ dominant peak frequency shifts toward lower frequencies in the θ-α band and higher frequencies in the β band, suggesting that frequency-domain EEG analysis holds promise for providing reliable biomarkers for NP [129]. Currently, EEG-related research remains limited. Existing results are derived primarily from intergroup comparisons, the causal relationships between EEG changes and pain onset and progression have not been clarified, and there is a lack of correlation analyses with brain anatomical features. These electrophysiological changes may be broadly distributed in pain-related brain regions which accounts for why non-invasive stimulation techniques aimed at specific nodes of brain networks can exert therapeutic effects such as interventions targeting the sensorimotor rhythm in the central region that can exert different effects by regulating frequency band ratios [130]. To advance the clinical application of EEG technology in pain assessment, it is essential to clarify the clinical correlations between the characteristics of each frequency band and the sensory, emotional, and cognitive components of pain, thereby ensuring its specificity, reliability, and relevance.

#### Structural imaging

Structural imaging reflects long-term pathological damage associated with the NP by quantifying changes in neural structure, mainly via ultrasound and MRI techniques. Ultrasound technology plays a crucial role in the diagnosis and treatment of neuromuscular diseases because of its cost-effectiveness, clinical accessibility, and technological advancements in high-resolution imaging. The application of high-resolution probes allows for precise alignment between the internal anatomical structure of nerves and ultrasound images while microvascular imaging technology facilitates the assessment of the neurovascular system which clearly visualizes the epineurial vascular network and some intraneurial vascularization characteristics [131, 132]. Ultrasound assessment metrics for nerve lesions include parameters such as the nerve cross-sectional area and nerve fascicle diameter [133–136]. For example, chronic immune-mediated neuropathy often presents with swollen nerve fascicles and hypoechogenicity in the acute/subacute phase, whereas in the chronic phase, it is characterized by nerve fascicle hyperechogenicity and a reduced diameter [137, 138]. Shear wave elastography (SWE) generates tissue elasticity maps by quantifying the shear wave propagation velocity. In studies on diabetic polyneuropathy, the nerve stiffness parameters measured via this technique are positively correlated with disease severity [139]. Although this technique is still in the exploratory phase, it has promising application potential in differentiating nerve lesion phases and evaluating treatment responses.

MRI delivers high-contrast and high-resolution images which clearly visualize neural structures and pathological changes while some of its sequences enable the quantification of specific structural parameters of nerves making MRI a promising imaging tool for diagnosing and monitoring NP [140, 141]. Using MRI technology, the association between brain morphology and pain sensitivity can be thoroughly explored. For example, consistent conclusions have been drawn regarding the correlation between certain brain regions including the precuneus and pain sensitivity, but the relationship between gray matter morphology and pain sensitivity exhibits two distinct outcomes including positive and negative correlations and this discrepancy may stem from heterogeneity in relationships across different brain regions [142–145]. Methodologically, studies have developed multivariate machine learning models to predict pain sensitivity by leveraging brain morphological features. In addition the clinical utility of brain morphological changes is further reflected in the development of candidate diagnostic biomarkers such as machine learning models based on gray matter density patterns in brain regions including the primary somatosensory cortex that can distinguish patients with chronic low back pain from healthy controls with 76% accuracy [146].

#### Functional and molecular imaging

Functional imaging (e.g., fMRI) and molecular imaging (e.g., PET) enable dynamic reflection of pathological activity associated with the NP and effective evaluation of therapeutic efficacy and prognostic outcomes. fMRI can distinguish patients with NP from healthy individuals by analyzing brain activation patterns and resting-state functional connectivity (rsFC). For example in patients with chronic low back pain the activation response in the nucleus accumbens significantly diminishes as pain stimuli gradually subside and the classification model developed on the basis of neural activity patterns in this region can completely distinguish patients with chronic low back pain from healthy controls [147] while a bimodal model that integrates brain activation features under painful and non-painful stimuli with NP-related symptoms achieves a sensitivity of 92% and a specificity of 94% [148] when differentiating between patients with fibromyalgia and healthy controls and the ToPS model developed on the basis of dynamic functional connectivity during persistent pain achieves classification accuracies of 71% and 73% in two independent datasets when distinguishing patients with chronic low back pain from healthy controls [149]. The identification of diagnostic biomarkers for NP disease subtypes is also a research focus. For example patients with migraine without aura and migraine with aura show significant differences in blood oxygen level-dependent responses induced by visual stimulation and visual system rsFC patterns and cluster analysis that integrates clinical measurement indicators and whole-brain functional connectivity data can identify two migraine subgroups that differ in headache frequency and self-rating depression scale scores with functional connectivity differences mainly concentrated in certain regions for example the amygdala [150–152]. Biomarkers for pain chronicity susceptibility or risk rely on longitudinal study designs. For example, increased functional connectivity strength within the prefrontal-striatal circuit (between the nucleus accumbens and medial prefrontal cortex) in patients with chronic low back pain can predict the chronic transformation of subacute low back pain after 1 year, and this result has been validated in an independent dataset [153]. Prognostic biomarkers can be used to predict outcomes for patients with chronic pain and for example rsFC strength between the left frontal lobe and left parietal lobe can serve as a predictor of 3-month disease improvement in patients with chronic pelvic pain syndrome [154]. Currently, although candidate biomarkers developed on the basis of brain imaging changes in patients with chronic pain have good sensitivity their specificity still requires verification in mixed datasets containing multiple pain types while biomarkers developed for specific pain states or treatment scenarios are difficult to apply to other types of chronic pain One approach to address this issue is to develop exclusive prediction models for each type of chronic pain.

TSPO and COX-2 are prominent biomarkers of neuroinflammation and they not only contribute to the pathological process of neuroinflammation but also serve as vital targets for inflammatory diagnosis. Techniques such as PET and single-photon emission computed tomography (SPECT) enable spatial localization, dynamic tracking of inflammatory lesions, monitoring of treatment responses, and prediction of clinical outcomes [155]. TSPO is a highly hydrophobic protein localized on the outer mitochondrial membrane that is highly expressed in steroid-synthesizing organs but in the resting state of the healthy brain TSPO expression in microglia is extremely low [156]. Under inflammatory conditions, TSPO expression is significantly upregulated, making it a prominent target for PET imaging. Acute nerve injury or neurodegenerative diseases can induce the activation of the proinflammatory phenotype of microglia and immunohistochemical staining combined with PET imaging studies have confirmed that in various animal models of neuroinflammation TSPO expression is increased and TSPO is spatially colocalized with reactive astrocytes and activated microglia [157–159]. COX-2 expression is induced by inflammatory and painful stimuli and it plays a dual role as a biomarker and an effector enzyme in conditions such as post-traumatic brain injury (a neurodegenerative-related disorder) but its specific mechanism of action in disease progression requires further verification [160–163].

Changes in the information processing mechanisms of the CNS are a critical foundation for maintaining abnormal pain perception in chronic pain. The core features of these networks include abnormal activity and connectivity patterns of multiple functional brain networks, with the brain amplifying sensory input signals [164–166]. This impact also extends to the autonomic nervous system (ANS) and studies have shown that high-frequency heart rate variability (HRV) is significantly reduced in patients with chronic pain. This finding suggests that weakened parasympathetic regulatory function may be closely associated with the onset and progression of chronic pain [167]. Given that brain imaging data and autonomic function indicators are expected to become objective quantitative tools for capturing pain experience, multivariate machine learning techniques have gained extensive attention in this field. These techniques extract features from clinically relevant data enabling computer algorithms to learn and develop predictive models that are then applied to the accurate prediction of disease states and objective evaluation of therapeutic effects [168]. For example, some studies have developed multivariate machine learning models that integrate CNS and ANS features for clinical pain state classification and pain intensity prediction. These models incorporate multimodal features such as rsFC patterns in the dorsal primary somatosensory cortex and the results confirm that this method can effectively classify pain states and predict clinical pain intensity [167, 169–171]. Other studies have developed multi-dimensional models that integrate brain function and genetic and psychological factors to systematically analyze the potential mechanisms of individual pain sensitivity. They reported that pain thresholds are more likely to be regulated by stable physiological factors (e.g., genotypes) whereas pain tolerance is more sensitive to psychological variables and further analysis demonstrated that brain network activity patterns play a vital role in the association between genetic and psychological factors and pain sensitivity with this effect being more significant in males. All the above studies highlight the significant potential of integrating multimodal neuroimaging data in the development of pain biomarkers [144, 172–174].

### Genetic biomarkers and epigenetic modifications

Genetic and most epigenetic biomarkers can be detected via peripheral blood genomic DNA or RNA extraction, which is minimally invasive. However, tissue-specific epigenetic modifications at the nerve or spinal cord level require invasive biopsy and are currently limited to preclinical research.

#### Genes related to genetic susceptibility

Genetic polymorphisms contribute to determining individual susceptibility to NP and differences in treatment efficacy by influencing enzyme activity, receptor function, or ion channel properties. The complexity of pain phenotypes arises from the combined effects of multiple factors, including interactions among multiple genes [175]. Currently, candidate genes of great interest include gene families encoding functional proteins (e.g., metabolic enzymes and transporters), specifically catechol-O-methyltransferase (COMT) and mu-opioid receptor 1 (OPRM1) [176]. COMT is a vital enzyme that catalyzes the degradation of catecholamines and modulates dopaminergic, adrenergic, and noradrenergic neurotransmission. Given the core role of catecholamines in pain conduction and regulation, polymorphisms and haplotypes of the COMT gene have garnered considerable attention in the research community [177–179]. The COMT gene has dozens of single nucleotide polymorphisms (SNPs) and is associated with a variety of mental and neurological disorders [180–182]. Studies have demonstrated that three allele combinations (i.e., haplotypes) consisting of four SNPs in the COMT gene are significantly associated with pain sensitivity, and these haplotypes are considered signature genetic features reflecting pain sensitivity [183, 184]. COMT polymorphisms and epigenetic factors jointly regulate COMT expression levels and enzyme activity, thereby altering the degradation rate of catecholamines, which in turn affects related neurotransmission processes and affects the pain matrix. Since these haplotypes have a significant impact on cross-modal pain thresholds and pressure pain thresholds, it is essential to jointly assess the synergistic effects of multiple COMT SNPs [185].

The OPRM1 gene is one of the most widely studied genes in the pharmacodynamics of opioid drugs and is relevant to multiple pain treatment scenarios. However, research findings on the correlation between the A118G polymorphism and the analgesic effects and opioid dose requirements remain contradictory. Some studies have reported no significant associations between the polymorphism and these opioid-related outcomes. Moreover, this polymorphism is associated with only chronic low back pain not other types of chronic pain [186–189]. In addition, the clinical impacts of the A118G polymorphism exhibit ethnic heterogeneity. G allele carriers in the Asian population require higher opioid doses to achieve the desired analgesic effect. This phenomenon has not been observed in the European population [190–192]. A study involving hospitalized patients compared the efficacy of five opioid drugs and the results revealed that G allele carriers presented a significantly smaller reduction in pain scores when treated with drugs such as hydromorphone while the therapeutic effects of methadone and tapentadol were not affected by this polymorphism which suggests that these two drugs may be more suitable for G allele carriers [193]. Therefore, research on OPRM1 genetic polymorphisms holds significant clinical value and is expected to provide a basis for the personalized development of opioid treatment regimens [194].

The occurrence of the NP is closely associated with changes in primary afferent nerve function and central nervous system sensitization. After nerve injury, the transection of peripheral axons of sensory neurons alters their gene transcription patterns, thereby leading to changes in the expression levels of nearly one-third of the genome [195–197]. These transcriptional changes involve the voltage-gated sodium channel family and these channels exhibit mislocalization after nerve injury due to abnormal axonal transport and structural changes in nodes of Ranvier [198, 199]. Among them, the Nav1.7 channel which is encoded by the SCN9A gene is specifically highly expressed in DRG sensory neurons and sympathetic ganglion neurons and serves as a core molecular component of human pain signal conduction. Its loss-of-function mutations result in congenital insensitivity to pain whereas its gain-of-function mutations are associated with a variety of painful diseases [200–204]. Studies involving patients with hereditary erythromelalgia have confirmed that excessive excitation of nociceptors represents the core pathogenesis of this disease [205, 206]. Currently, multiple studies have investigated the electrophysiological characteristics of Nav1.7 mutants at the cellular level, clarifying the associations between mutation nature, mutation sites, channel function, and clinical phenotypes and improved gene sequencing technologies and bioinformatics analyses have facilitated the identification of disease-related mutation sites, thereby providing an important basis for elucidating ion channel functions [201, 203, 207].

The TRPV1 channel is highly expressed in sensory neurons that specialize in detecting noxious stimuli and is involved in pain perception and transmission [208]. Although more than 1,000 genetic variants of the TRPV1 channel have been reported, the functions of most of these variants remain unclear. Studies have demonstrated that blocking the TRPV1 channel can reduce pain responses, but most TRPV1 antagonists have adverse effects [209]. In vitro site-directed mutagenesis studies have demonstrated that specific point mutations can selectively inhibit the activation of the TRPV1 channel while preserving its heat-sensing function, suggesting that “mode-selective antagonists” may be a more promising pain treatment strategy [210, 211]. The latest research further demonstrated that residue K710 of the TRPV1 channel is a vital node for pain gating and mutations at this residue can selectively inhibit chemical pain perception while retaining heat perception and cell protection functions thereby providing theoretical support for the development of new analgesic drugs [212].

From a therapeutic perspective, TRPV1-targeted interventions include both antagonists and high-dose agonists, with distinct clinical applications. While TRPV1 antagonists have been investigated for systemic analgesia, their development has been hampered by adverse effects such as impaired thermoregulation. In contrast, high-dose topical capsaicin (a potent TRPV1 agonist) has established clinical value in neuropathic pain management. Prolonged activation of TRPV1 by high-concentration capsaicin induces reversible defunctionalization of nociceptors, including depletion of neuropeptides, reduction of nerve terminal excitability, and temporary degeneration of epidermal nerve fibers, thereby producing long-lasting analgesia. High-dose capsaicin patches have been approved for the management of postherpetic neuralgia and painful diabetic peripheral neuropathy, and are recommended as second- to third-line therapeutic agents in numerous clinical practice guidelines. This agonist-based therapeutic strategy provides an alternative approach to targeting TRPV1, complementing the development of selective antagonists.

#### Dynamic regulation of epigenetic modifications

Epigenetic modifications modulate the expression of pain-related genes by altering chromatin structure or RNA function, and their dynamic changes are closely associated with the progression of the NP. Epigenetics focuses on gene expression regulatory mechanisms that do not rely on changes in nucleotide sequences. This regulation is based on molecular-level changes in chromatin structure and its regulatory process is jointly influenced by endogenous and exogenous factors primarily manifesting as covalent DNA methylations or post-translational histone modifications ultimately leading to the transcriptional upregulation or downregulation of gene expression [213]. Vital proteins involved in DNA methylation and demethylation processes are localized in the DRG and three DNA methyltransferases including DNMT1, DNMT3a and DNMT3b are stably expressed in the DRG under physiological conditions [214–217]. A study involving a rat sciatic nerve injury model demonstrated that on the 14th day after nerve injury, global DNA methylation levels in lumbar spinal cord tissues were significantly increased, and the expression of methyl-CpG-binding protein 2 (MeCP2) was increased. Intrathecal injection of 5-azacytidine (a non-selective DNA methyltransferase inhibitor) inhibited these changes and alleviated mechanical allodynia and thermal hyperalgesia in rats, suggesting that nerve injury may induce epigenetic silencing of certain anti-nociceptive genes via DNA methylation mechanisms [214, 218]. In the SNI model the expression levels of DNA methyltransferases and MeCP2 mRNA in spinal dorsal horn tissues were downregulated and MeCP2 expression in the ipsilateral injured DRG was also significantly downregulated simultaneously the level of 5-hydroxymethylcytosine a marker of DNA demethylation in the ipsilateral DRG was significantly increased and TET3 protein expression was increased and TET1 and TET2 expression remained unchanged these findings indicate that nerve injury can also induce DNA hypomethylation [219]. In the paclitaxel-induced neuropathy model, no significant changes in DNA methylation levels were observed in DRG tissues however in the streptozotocin-induced diabetic mouse model 376 hypermethylated genes and 336 hypomethylated genes were identified at CpG sites in the DRG promoter region [220, 221]. Genome-wide analysis of human subjects revealed that the DNA methylation pattern of blood cells in patients with NP was significantly different from that in healthy controls and patients with nociceptive pain [222].

Histone acetylation primarily occurs at lysine residues in the amino-terminal tails of histones [223, 224]. Histone deacetylases (HDACs), a class of epigenetic erasers, strip acetyl groups from histone tails, thereby promoting the formation of heterochromatin. In the rat DRG, the expression of HDAC1, HDAC2, HDAC4, and HDAC5 has been detected, among which HDAC2, HDAC4, and HDAC5 are primarily localized in neurons [225]. In different nerve injury models, the expression of these HDAC subtypes exhibits specific changes. For instance, in the sciatic nerve injury model the expression levels of HDAC2, HDAC4 and HDAC5 are significantly upregulated. Moreover, HDAC2 is highly expressed in neurons, slightly expressed in astrocytes and undetectable in microglia [226]. In the SNI model the expression of HDAC2 in spinal astrocytes was significantly increased 7 days post-injury whereas no significant changes were observed in neurons and simultaneously the overall expression of HDAC1 and HDAC2 in spinal cord tissues was upregulated whereas HDAC3 expression remained stable [227, 228]. Intervention studies on HDAC1 demonstrated that intrathecal injection of the selective HDAC1 inhibitor LG325 could reverse spinal cord HDAC1 upregulation and alleviate mechanical allodynia and that oral administration of zingiberene (a selective HDAC1 inhibitor) could exert effects consistent with those of LG325 namely reversing HDAC1 upregulation alleviating mechanical allodynia and further alleviating thermal hyperalgesia [228, 229]. SIRT1 is a class III NAD^+^-dependent HDAC. In a rat CCI model, when thermal hyperalgesia and mechanical allodynia occur, SIRT1 expression levels in spinal cord tissues are significantly downregulated, and the acetylation levels of H3 and H4 histones are significantly increased [230, 231]. Intrathecal injection of the SIRT1 activator resveratrol could delay the onset of hyperalgesia and allodynia. It could also reverse histone hyperacetylation. A more potent SIRT1 activator named SRT1720 produced similar outcomes. Meanwhile, SIRT1 inhibitors could block the analgesic effect of resveratrol [232]. Histone methylation is another form of post-translational histone modification that primarily occurs at lysine or arginine residues in histone tails [224, 233]. In the sciatic nerve ligation model, the G9a/GLP complex mediates H3K9me2 methylation. It thereby silences the expression of potassium channel genes like Kv1.2 and Kv4.2. It also silences the expression of opioid receptor genes. This process finally leads to increased neuronal excitability [234, 235]. Meanwhile, EZH2 mediates H3K27me3 methylation. It promotes the activation of spinal microglia. It also enhances the expression of inflammatory factors [236].

Post-transcriptional RNA modifications modulate the expression of target genes and are involved in regulating various neurobiological functions. In-depth analysis of how these modifications regulate the expression of pain-related genes is expected to identify novel NP biomarkers [237, 238]. Currently, it is well established that RNA modifications including N6-methyladenosine (m6A), 5-methylcytosine (m5C) and N4-acetylcytidine (ac4C) play vital roles in regulating RNA stability, subcellular localization and translational efficiency [239–242]. And notably, the regulatory mechanisms of m6A and ac4C in the NP have been studied more systematically. m6A methylation is the most abundant RNA modification in eukaryotes and is involved in the onset and maintenance of the NP. Its modification sites are typically located within the consensus RRACH motif and are enriched near stop codons and 3’ UTRs [243, 244]. This modification is dynamically and reversibly regulated by methyltransferases and demethylases. In turn, the fate of transcripts is ultimately determined by the recognition of target RNAs by m6A-binding proteins [245]. When the dynamic balance between methyltransferases and demethylases is disrupted, the m6A levels of RNAs are altered, which in turn affects the stability and translational efficiency of pain-related mRNAs post nerve injury, ultimately inducing NP. METTL3 participates in pain progression through multiple pathways. First, it interacts with the DGCR8 protein to promote the processing of pri-miR-355-3p, and this effect enhances the chronic pain phenotype in NP models. Additionally, it inhibits miR-100 maturation, and this action stabilizes the BDNF protein and exacerbates NP. Furthermore, it interacts with the YTHDF2 protein to downregulate the expression of the lncRNA D26496, and this downregulation promotes pain development by inhibiting Schwann cell proliferation. Moreover, it stabilizes TET1 with the help of the YTHDF2 protein, and this stabilization exacerbates inflammatory pain by regulating DNA demethylation. On the other hand, FTO regulates pain primarily through two pathways. One of these pathways is that it enhances G9a expression in the DRG, and this enhancement increases neuronal excitability by inhibiting OPRM1. The other pathway is that it stabilizes the TLR4 and MMP24 proteins, and this stabilization promotes the phosphorylation of extracellular signal-regulated kinases in the spinal cord and thereby induces hyperalgesia [234, 246–252]. ac4C is a widespread and highly conserved post-transcriptional RNA modification and is associated with the onset, progression, and prognosis of various diseases [253, 254]. In the SNI model, ac4C modification in the spinal dorsal horn regulates VEGFA expression via a dual regulatory mechanism, ultimately mediating pain-related behaviors. First, a direct increase in ac4C levels in the spinal dorsal horn enhances the translational efficiency of VEGFA mRNA, thereby increasing its protein expression. Second, the binding capacity of the HNRNPK protein to VEGFA mRNA is significantly increased and this increase further promotes the recruitment of NAT10 which is a vital ac4C modification enzyme and the subsequent enrichment of NAT10 further raises the ac4C modification levels of VEGFA mRNA and in doing so it forms a positive feedback loop. Together, these two regulatory pathways drive significant upregulation of VEGFA expression, inducing central sensitization in the spinal dorsal horn and ultimately triggering pain-related behaviors in the SNI model [255] (Table 1).

**Table 1:** Comparison of applications and limitations of four types of biomarkers in neuropathic pain

Type	Applications	Examples	Limitations	Detection Accessibility	Future Combination Potential
Biochemical	Directly reflect molecular pathological processes of NP (e.g., neuroinflammation, oxidative stress). Enable early screening of high-risk NP populations (e.g., PDN, PHN). Assist in evaluating disease progression and treatment response.	Neuroinflammation-related: TNF-α, IL-1β, TLR4 (PDN/PHN). Metabolism/oxidative stress-related: AGEs, MG (PDN). Non-coding RNA: lncRNA LINC01119 (PHN), miR-29a/500 (lingual nerve neuroma).	Single biomarker lacks sufficient sensitivity and specificity (e.g., IL-6 levels show conflicting correlations in PDN). Humoral detection (blood, CSF) may be interfered by systemic factors (e.g., other inflammatory diseases). Difficulty in distinguishing NP subtypes due to overlapping molecular signatures.	Mostly non-invasive (peripheral venous blood sampling). CSF detection is minimally invasive and suitable for routine clinical testing.	Combine with imaging biomarkers (e.g., TNF-α + PET TSPO) to localize inflammation and quantify severity. Integrate with genetic biomarkers (e.g., lncRNA LINC01119 + OPRM1 A118G) to improve subtype diagnosis and treatment guidance.
Electrophysiological	Quantify abnormal nerve function objectively (e.g., conduction velocity, small-fiber integrity). Detect small-fiber lesions and spinothalamic tract abnormalities (key for early NP). Predict postoperative NP risk via intraoperative monitoring.	Small-fiber assessment: Laser-EPs, CHEPs (PHN/CNP). Large-fiber/central conduction: NCS, SEPs (peripheral large myelinated fibers). Central sensitization: EEG.	NCS/SEPs yield normal results in patients with isolated small-fiber or spinothalamic lesions (miss high-risk groups). EEG research lacks clarification of causal relationship with pain onset; limited correlation with brain anatomy. Requires painful stimuli for testing, leading to stronger correlation with evoked pain than spontaneous pain.	Fully non-invasive; widely available in outpatient and inpatient settings, suitable for repeated follow-up.	Combine with structural imaging (e.g., laser-EPs + MRI spinal cord) to confirm small-fiber injury and central lesions. Integrate with EEG + fMRI (resting-state functional connectivity) to link electrophysiological activity with brain functional remodeling.
Imaging	Visualize neural structural remodeling (e.g., spinal glial scar, brain gray matter atrophy). Distinguish NP subtypes via functional/molecular patterns (e.g., PHN vs. CNP). Predict pain chronicity and treatment efficacy (e.g., prefrontal-striatal connectivity).	Structural: MRI (spinal dorsal horn neuronal proliferation, brain gray matter density), ultrasound SWE (diabetic polyneuropathy nerve stiffness). Functional: fMRI (nucleus accumbens activation, resting-state functional connectivity). Molecular: PET (TSPO for microglial activation, COX-2 for inflammation).	fMRI biomarkers lack specificity across multiple pain types (needs verification in mixed pain datasets). PET has high cost and radiation exposure, limiting long-term follow-up. Ultrasound SWE is still exploratory; standardization of stiffness parameters is lacking.	Ultrasound and conventional MRI are non-invasive and widely accessible. PET is non-invasive but has higher cost and radiation exposure.	Combine with electrophysiological biomarkers (e.g., fMRI + EEG) to integrate functional and electrical activity. Integrate with genetic biomarkers (e.g., PET TSPO + COMT polymorphism) to explain individual differences in neuroinflammation and pain sensitivity.
Genetic/Epigenetic	Clarify individual susceptibility to NP (e.g., COMT haplotypes for pain sensitivity). Guide personalized medication (e.g., OPRM1 A118G for opioid selection). Reveal epigenetic regulatory mechanisms of NP progression (e.g., m6A modification).	Genetic: COMT Val158Met, OPRM1 A118G, SCN9A (Nav1.7), TRPV1 K710. Epigenetic: DNA methylation (DNMT3a), histone modification (HDAC1/SIRT1), RNA modification (METTL3/FTO, NAT10/ac4C).	NP heterogeneity restricts cross-etiology universality (e.g., OPRM1 A118G correlates with chronic low back pain). Epigenetic modifications are dynamic; difficult to capture stable signatures for diagnosis. Most genetic variants (e.g., TRPV1 variants) lack clear clinical translation due to unclear function.	Peripheral blood genotyping is minimally invasive; tissue-specific epigenetic detection requires biopsy and is limited to research.	Combine with biochemical biomarkers (e.g., OPRM1 A118G + IL-6) to link genetic susceptibility with inflammatory status. Integrate multi-omics (genomics + epigenomics + transcriptomics) with AI to screen composite biomarkers for NP subtype prediction.

## Translational challenges and functional classification of biomarkers

### Limitations of preclinical animal models in biomarker translation

A critical challenge in NP biomarker translation stems from inherent differences between preclinical animal models and clinical patient populations. Most animal models of NP rely on controlled nerve injury (e.g., CCI, SNI, spinal nerve ligation) and measure pain responses via evoked reflexes (mechanical allodynia, thermal hyperalgesia) triggered by external stimuli. These models primarily capture provoked pain and reflexive nociceptive behaviors, but cannot fully recapitulate the core clinical features of human NP, particularly continuous spontaneous pain, paroxysmal spontaneous pain, and the emotional-cognitive dimensions of chronic pain.

This methodological discrepancy has important implications for biomarker validation. Biomarkers strongly associated with evoked reflexive pain in animals may not correlate well with spontaneous pain intensity in patients. For example, many inflammatory mediators and ion channel biomarkers identified in rodent evoked pain models show inconsistent correlations with clinical pain scores. Furthermore, animal models typically involve young, genetically homogeneous animals with single-etiology nerve injury, whereas clinical NP patients are often older, present with multiple comorbidities, and exhibit heterogeneous etiologies and sensory phenotypes. These differences contribute to the high failure rate of biomarker translation from bench to bedside. To systematically elaborate the translational advancement of various candidate biomarkers and bridge the disconnect between preclinical observations and clinical verification, representative NP biomarkers are summarized in Table 2 based on their discovery origins and human validation statuses.

**Table 2:** Classification of neuropathic pain biomarkers by discovery source and human validation status

Biomarker Category	Specific Biomarker	Discovery & Validation Status	Corresponding NP Subtype	Detection Method
Biochemical	TNF-α, IL-1β	Animal-identified, human-validated	PDN, PHN, CNP	Serum/CSF ELISA
	AGEs, methylglyoxal	Animal-identified, human-validated	PDN	Serum biochemical assay
	lncRNA LINC01119	Animal-identified, human-validated	PHN, traumatic NP	Serum qPCR
	MCP-1	Animal-identified, human-validated	Traumatic NP	Serum/CSF
	Mitochondrial ETC complex proteins	Animal-only (limited human correlative data)	Multiple NP subtypes	DRG/spinal cord tissue
Electrophysiological	Laser-EPs, CHEPs	Human-identified and validated	Small-fiber neuropathy, PHN, CNP	Cutaneous stimulation + EEG recording
	WDR neuron firing patterns	Animal-only	Multiple NP subtypes	In vivo extracellular recording
	EEG θ-low α frequency band changes	Human-identified and validated	Chronic NP	Scalp EEG
Imaging	TSPO PET signal	Animal-identified, human-validated	CNP, chronic NP	PET imaging
	Gray matter density changes in pain matrix	Human-identified and validated	Chronic NP	Structural MRI
	Resting-state functional connectivity patterns	Human-identified and validated	Multiple NP subtypes	fMRI
Genetic/Epigenetic	SCN9A (Nav1.7) mutations	Human-identified and validated	Hereditary erythromelalgia, small-fiber neuropathy	Genome sequencing
	COMT haplotypes	Human-identified and validated	Multiple chronic pain conditions	Genotyping
	OPRM1 A118G polymorphism	Human-identified and validated	Chronic low back pain, postoperative pain	Genotyping
	G9a-mediated H3K9me2	Animal-only	Traumatic NP	DRG/spinal cord tissue assay
	m6A regulators (METTL3, FTO)	Animal-identified, preliminary human validation	Traumatic NP	Tissue/serum assay
	ac4C modification of VEGFA	Animal-only	Traumatic NP	Spinal cord tissue assay

Future preclinical research should incorporate more spontaneous pain assessment paradigms (e.g., conditioned place preference, operant behavioral tests) and establish animal models that better mimic clinical comorbidities and chronic pain progression, to improve the predictive validity of candidate biomarkers.

### Functional classification of biomarkers: diagnostic vs. predictive

From a clinical application perspective, NP biomarkers can be functionally divided into two major categories that each serve distinct clinical purposes. The first category comprises diagnostic and differential diagnostic biomarkers, and the second comprises treatment response predictive biomarkers.

Diagnostic and differential diagnostic biomarkers are used to confirm the presence of neuropathic pain, distinguish NP from nociceptive or psychogenic pain, and identify etiological subtypes and sensory phenotypes. Core diagnostic biomarkers cover multiple modalities. Electrophysiological biomarkers address different diagnostic needs. Laser-EPs and CHEPs confirm small-fiber injury, NCS supports diagnosis of large-fiber neuropathy, and EEG patterns assist assessment of central NP. Imaging biomarkers fulfill distinct diagnostic roles. TSPO PET localizes neuroinflammation, structural MRI identifies nerve and spinal cord lesions, and fMRI connectivity patterns help differentiate NP subtypes. Biochemical biomarkers consist of etiology-specific molecules. Examples include AGEs for PDN and VZV-specific immune markers for PHN. Genetic biomarkers include variants such as SCN9A mutations associated with hereditary channelopathies.

Treatment response predictive biomarkers are used to guide personalized therapy selection, predict efficacy and adverse effects, and monitor treatment response. Key predictive biomarkers span multiple categories. Genetic biomarkers inform treatment selection. The OPRM1 A118G polymorphism guides opioid selection, and COMT haplotypes help predict catecholamine-related analgesic response. Biochemical biomarkers support efficacy forecasting. Baseline TNF-α and IL-1β levels predict response to anti-inflammatory therapies, and baseline methylglyoxal levels indicate likely response to anti-glycation treatments. Imaging biomarkers carry predictive value. Baseline TSPO expression level predicts response to glia-targeted therapies, and resting-state connectivity patterns forecast neuromodulation treatment outcomes. Epigenetic biomarkers feature specific RNA modification signatures that predict response to epigenetic-targeted interventions.

This functional classification framework helps clinicians select appropriate biomarker panels for different clinical scenarios. Screening and diagnosis draw primarily on non-invasive diagnostic biomarkers, while personalized treatment planning integrates predictive biomarkers to optimize therapeutic outcomes.

## Conclusion and outlook

**Fig. 2:**
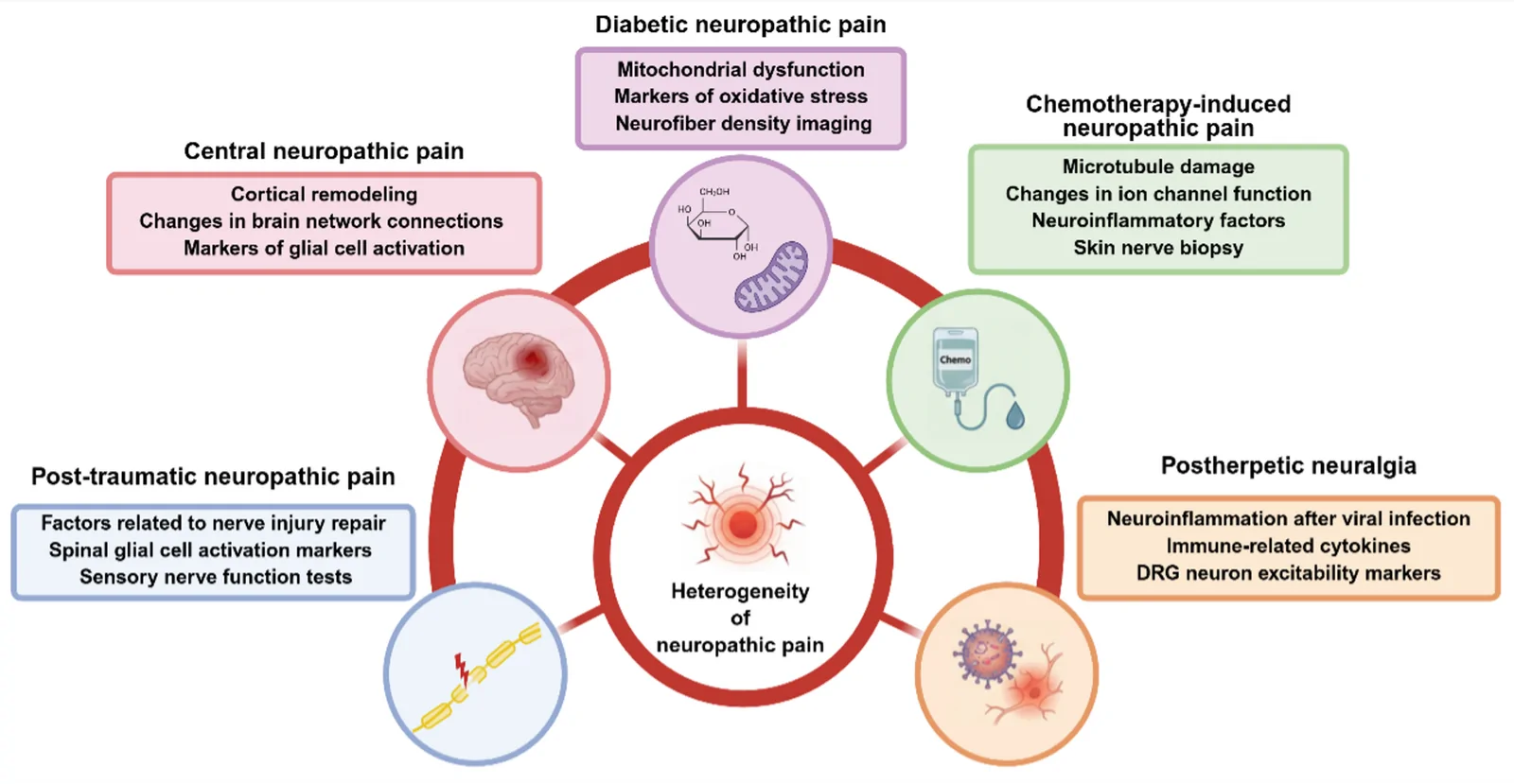
Heterogeneity of neuropathic pain and a biomarker panel-based strategy for subtyping. This figure depicts the marked clinical and pathological heterogeneity of neuropathic pain and further illustrates how mapping subtype-specific pathophysiological mechanisms to corresponding biomarker panels can address the key challenges of standardized neuropathic pain diagnosis and treatment

There remain several unaddressed challenges in the current diagnosis and treatment of NP. For instance, contemporary clinical diagnosis is primarily based on subjective symptoms and neurofunctional assessments, resulting in difficulties in early detection and delayed assessment of therapeutic efficacy. Therefore, more robust strategies are needed to achieve precision diagnosis and personalized treatment. This review systematically analyzes the pathological mechanisms of NP, categorizes biomarkers, and synthesizes research findings across multiple platforms, with the goal of developing an objective biomarker system to address the aforementioned challenges. At the mechanistic level, pathological plastic changes in the sensory nervous system are the primary drivers of NP development and progression, and NP with distinct etiologies is also associated with distinct pathological pathways. At the biomarker level, diverse classes of biomarkers hold promising clinical applicability. First, biochemical biomarkers effectively reflect the molecular pathologies of NP. In addition, electrophysiological biomarkers offer quantifiable insights into neurofunctional abnormalities. Furthermore, imaging biomarkers aid in uncovering anatomical pathologies and pathological functional plasticity of the nervous system. Also, genetic and epigenetic biomarkers elucidate factors linked to pain susceptibility and the role of epigenetic regulation (Fig. 2).

**Fig. 3:**
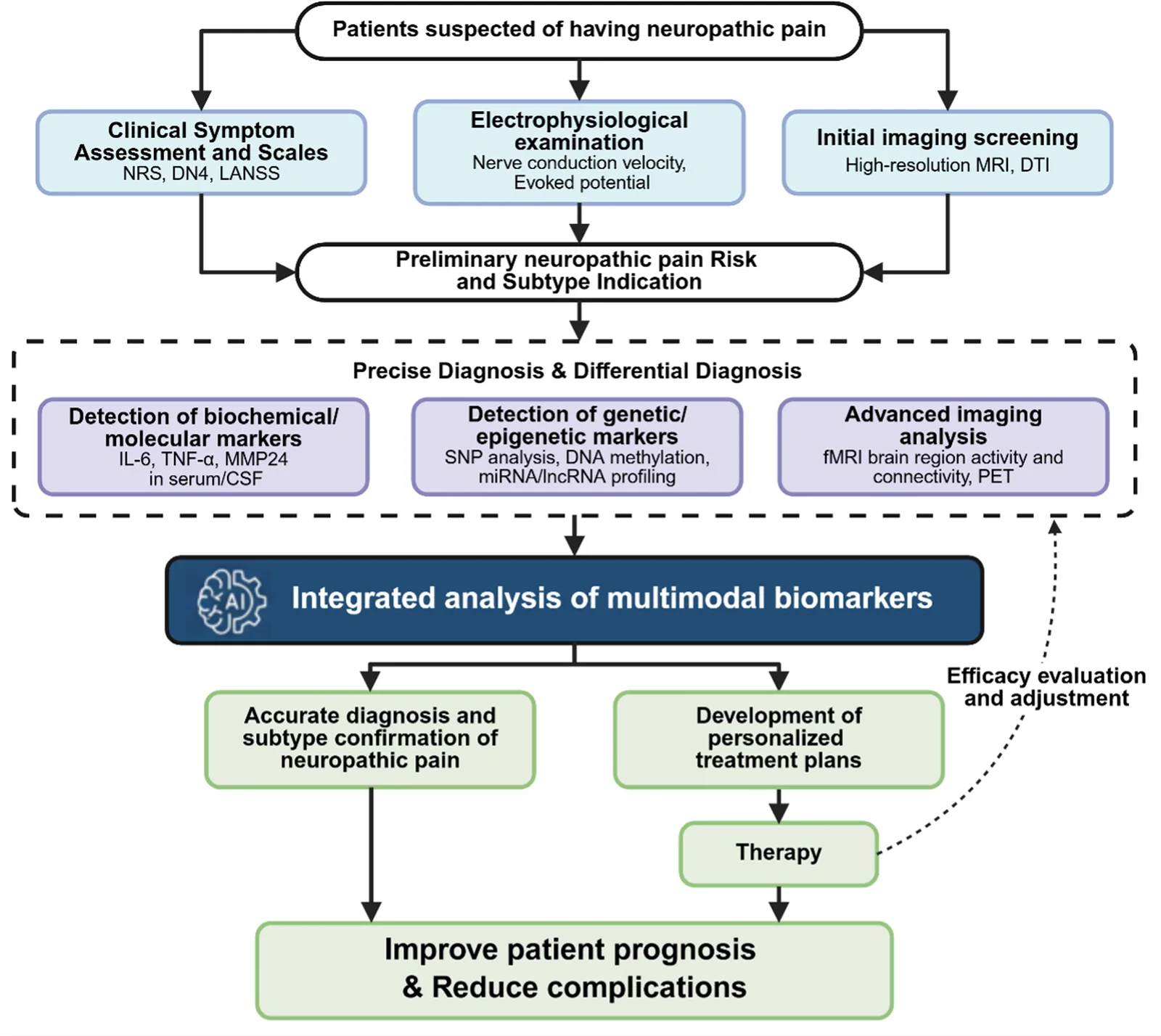
Clinical application flowchart for biomarker-based diagnosis and assessment in the clinical management of neuropathic pain. This flowchart outlines a sequential multimodal clinical pathway for neuropathic pain that encompasses preliminary screening with clinical scales (including DN4, LANSS and NRS), electrophysiological examination, initial imaging screening, precise diagnosis via multimodal biomarker detection, personalized therapy development, efficacy evaluation and prognostic optimization, while placing strong emphasis on the integration of biomarker-driven assessments

The above-mentioned conclusions not only confirm that NP biomarker research can be promoted by making multi-dimensional integrated analyses, but give new insights into the better understanding of NP pathological mechanisms (e.g., distinguishing specific and common pathological pathways of NP subtypes), and the preliminary establishment of objective biomarker systems. The above-mentioned conclusions deal with the current deficiency of NP specific and objective giving diagnoses in clinical medicine, and pave the way for the alienation from the model of empirical medicine of therapy and diagnosis. Specifically, the specific indicative value of serum pro-inflammatory factors (among biochemical biomarkers) in relation to inflammation induced by PHN virus, and the PDN-related immune system disorders, the ability of the laser EPs (among electrophysiological biomarkers) to indicate objective detection of small-fiber lesions, the indicative value of f-MRI functional connection patterns (among imaging biomarkers) in relation to the NP subtypes, and the guiding value of genetic biomarkers for personalizing the drug effect of opioids (such as OPRM1 A118G polymorphism indicates that methadone is advisable for G allele carriers) are results which all indicate that an enormous amount of guiding value is to be found in the multiplicity of the biomarkers to show the high-risk NP populations, the procedure of early diagnosis, the prediction of drug effect and an individualized treatment strategy. Furthermore, technical support is given for enhancing the clinical value of the biomarkers by the use of machine learning programs in relation to biomarker detection and integrated analysis (e.g., the development of classification models on the basis of imaging properties) (Fig. 3; Table 3).

**Table 3:** Neuropathic pain targeted therapy and biomarker guidance

Therapy	Target	Biomarker Guidance Value	Preclinical/Clinical Research Stage	References
Diagnostic & Subtyping Biomarkers	Small-fiber integrity, neuroinflammation, ion channel function	Laser-EPs/CHEPs: confirm small-fiber injury and distinguish irritable vs. deafferentation phenotypes. TSPO PET: localize neuroinflammation and differentiate central vs. peripheral NP. AGEs/VZV immune markers: etiological subtyping of PDN vs. PHN.	Clinical validation (routine use in specialized pain centers)	[109–111, 155–159]
Targeted Inhibition of Neuroinflammation	TLR4, TNF-α, IL-1β	TLR4: High expression in monocytes of PDN patients (5-fold vs. diabetic patients without neuropathy) guides TLR4 inhibitor selection to block the “inflammation-damage” cycle. TNF-α: Serum levels in PDN patients correlate with pain intensity/nerve demyelination, guiding TNF-α inhibition to improve nerve function. IL-1β: Elevated mRNA/protein in PDN serum/nerve tissues guides IL-1β antagonists to reduce neuronal dysfunction.	TLR4 inhibitors: Preclinical (alleviate inflammation/pain in NP models). TNF-α inhibitors: Clinical research (significantly improve nerve conduction velocity in PDN). IL-1β antagonists: Preclinical (reduce hyperalgesia in NP models).	[30–36]
Intervention in Oxidative Stress/Metabolic Damage	AGEs-RAGE, MG-TRPA1, Mitochondrial ETC (Complexes I/III/IV)	AGEs: Elevated in PDN nerve tissues/serum guides RAGE antagonists to inhibit glycotoxicity and TRPV1-mediated calcium signaling. MG: Elevated plasma in type 2 diabetic patients (neuropathy risk factor) guides TRPA1 inhibitors to block neuronal excitability. Mitochondrial ETC: Reduced OCR in DRG neurons (pain models) guides ETC activators to restore respiratory function.	AGEs-RAGE antagonists: Preclinical (prevent small-fiber neuropathy in diabetic models). MG-TRPA1 inhibitors: Preclinical (alleviate mechanical/thermal hyperalgesia). ETC activators (Dichloroacetate): Preclinical (restore bioenergetics and reduce pain).	[53–64, 67–79]
Targeted Regulation of Non-coding RNAs	lncRNA LINC01119, miR-29a/miR-500	lncRNA LINC01119: Significantly elevated serum in PHN patients guides intrathecal shRNA to silence its expression (stabilizes BDNF). miR-29a/miR-500: Serum levels positively correlated with pain VAS scores in lingual nerve neuroma patients, guiding miRNA mimics/inhibitors to regulate DRG neuron excitability.	lncRNA LINC01119 shRNA: Preclinical (alleviates pain in SNI rat models). miRNA regulators: Preclinical to Phase II (modulate pain-related gene expression in NP).	[93, 103]
Intervention in Epigenetic Modifications	METTL3 (m6A), FTO (m6A), HDAC1, SIRT1, NAT10 (ac4C)	METTL3/FTO: Abnormal expression in NP models (METTL3 upregulates BDNF; FTO stabilizes TLR4) guides m6A modifiers to restore RNA methylation balance. HDAC1: Upregulated in SNI spinal astrocytes guides selective inhibitors (LG325/zingiberene) to reverse histone deacetylation. SIRT1: Downregulated in CCI models guides activators (resveratrol) to reduce histone hyperacetylation. NAT10: Enriched in SNI spinal dorsal horn guides ac4C inhibitors to block VEGFA positive feedback.	m6A modifiers (METTL3/FTO inhibitors): Preclinical. HDAC1 inhibitors: Preclinical (alleviate mechanical allodynia in SNI models). SIRT1 activators: Preclinical (delay hyperalgesia onset in CCI models). NAT10 inhibitors: Preclinical (reduce VEGFA-mediated central sensitization).	[228–232, 246–252, 255]
Regulation of Ion Channel Function	SCN9A (Nav1.7), TRPV1 (K710)	SCN9A (Nav1.7): Gain-of-function mutations in hereditary erythromelalgia (excessive nociceptor excitation) guide Nav1.7 inhibitors to block ectopic discharge. TRPV1 (K710): K710 residue (pain gating node) guides “mode-selective antagonists” to inhibit chemical pain while retaining heat-sensing function.	Nav1.7 inhibitors: Preclinical to Phase II (reduce pain in erythromelalgia models). TRPV1 mode-selective antagonists: Preclinical (site-directed mutagenesis confirms selective pain inhibition).	[200–207, 212]
Personalized Opioid Receptor Therapy	OPRM1 (µ-Opioid Receptor)	OPRM1 A118G polymorphism: Ethnic heterogeneity (Asian G allele carriers require higher opioid doses) guides opioid selection (methadone/tapentadol are effective for G allele carriers; hydromorphone efficacy is reduced).	Clinical research (hospitalized patients: verified that OPRM1 genotype predicts opioid efficacy; methadone/tapentadol are preferred for G allele carriers).	[190–193]

However, current application of biomarkers remains constrained by multiple limitations. For instance, a single biomarker often lacks specificity and sensitivity. The etiological heterogeneity of NP is such as to limit most biomarkers in their universality of application across the different etiological types. Furthermore, the timescale for clinical translation of these biomarkers is still lengthy. In particular, the translational gap between preclinical animal findings and clinical application remains a core bottleneck, as most candidate biomarkers discovered in animal models of evoked pain have not been validated in patient populations with spontaneous pain. In future research, multicenter, large-cohort databases could be established to mitigate the impact of disease heterogeneity. Leveraging AI-enabled integrated analysis of multi-omics data will facilitate the identification of composite biomarkers with enhanced performance. Investigating precision intervention strategies targeting RNA modifications will promote the development of targeted therapeutic approaches. Developing predictive models for distinct NP subtypes will facilitate the integration of basic science and clinical practice, thereby accelerating the clinical translation timeline. Collectively, these cutting-edge initiatives will refine the precision diagnosis and treatment system for NP, harness the potential of data integration, and enable precise therapeutic intervention. In the future, it is anticipated that the expanding research focus on the validation and clinical translation of NP biomarkers will facilitate the transition of NP diagnostic and therapeutic approaches and thereby improve patients’ quality of life.

## Data Availability

No datasets were generated or analysed during the current study.

## Abbreviations

3’UTRs: 3’untranslated regions
ac4C: N4-acetylcytidine
AGEs: Advanced Glycation End products
ANS: Autonomic Nervous System
CCI: Chronic Constriction Injury
CFA: Freund’s adjuvant
CHEPs: Contact Heat-evoked Potentials
CNP: Central Neuropathic Pain
CNS: Central nervous system
COMT: Catechol-O-methyltransferase
COX-2: Cyclooxygenase-2
EEG: Electroencephalogram
fMRI: Functional Magnetic Resonance Imaging
H₂O₂: Hydrogen Peroxide
HDAC: Histone Deacetylase
HRV: Heart Rate Variability
laser-EPs: Laser-evoked Potentials
lncRNA: Long non-coding RNA
m5C: 5 – methyl cytosine
m6A: N6 – methyl adenosine
MeCP2: Methyl-CpG-Binding Protein 2
mETC: Mitochondrial Electron Transport Chain
MG: Methylglyoxal
microRNA: miRNA
mtROS: Mitochondrial Reactive Oxygen Species
NCS: Nerve Conduction Study
NP: Neuropathic Pain
NPSI: Neuropathic Pain Symptom Inventory
O₂⁻: Superoxide Anion
OCR: Oxygen Consumption Rate
OH⁻: Hydroxyl Radicals
OPRM1: Mu-Opioid Receptor 1
PDN: Peripheral Diabetic Neuropathy
PET: Positron Emission Tomography
PHN: Postherpetic Neuralgia
PKC: Protein Kinase C
PNS: Peripheral nervous system
ROS: Reactive Oxygen Species
rsFC: Resting-state Functional Connectivity
SEPs: Somatosensory Evoked Potentials
shRNA: Short Hairpin RNA
SNI: Selective Injury of the Sciatic Nerve Branches
SNP: Single Nucleotide Polymorphism
SPECT: Single-photon Emission Computed Tomography
SSRs: Sympathetic Skin Responses
SWE: Shear Wave Elastography
TCA: Tricarboxylic Acid
TSPO: Translocator Protein
VAS: Visual Analog Scale
VZV: Varicella-Zoster Virus
WDR: Wide Dynamic Range
